# Wicking: A Rapid Method for Manually Inserting Ion Channels into Planar Lipid Bilayers

**DOI:** 10.1371/journal.pone.0060836

**Published:** 2013-05-23

**Authors:** Justin A. Costa, Dac A. Nguyen, Edgar Leal-Pinto, Ronald E. Gordon, Basil Hanss

**Affiliations:** 1 Division of Nephrology, Department of Medicine and Graduate School of Biomedical Sciences, The Icahn School of Medicine at Mount Sinai, New York, New York, United States of America; 2 Department of Physiology and Biophysics, Virginia Commonwealth University, Richmond, Virginia, United States of America; 3 Department of Pathology, The Icahn School of Medicine at Mount Sinai, New York, New York, United States of America; 4 NIH Medical Scientist Training Program, The Mount Sinai School of Medicine, New York, New York, United States of America; 5 Department of Systems Biology of Disease and Therapeutics, Graduate School of Biomedical Sciences, The Icahn School of Medicine at Mount Sinai, New York, New York, United States of America; Consejo Superior de Investigaciones Cientificas, Spain

## Abstract

The planar lipid bilayer technique has a distinguished history in electrophysiology but is arguably the most technically difficult and time-consuming method in the field. Behind this is a lack of experimental consistency between laboratories, the challenges associated with painting unilamellar bilayers, and the reconstitution of ion channels into them. While there has be a trend towards automation of this technique, there remain many instances where manual bilayer formation and subsequent membrane protein insertion is both required and advantageous. We have developed a comprehensive method, which we have termed “wicking”, that greatly simplifies many experimental aspects of the lipid bilayer system. Wicking allows one to manually insert ion channels into planar lipid bilayers in a matter of seconds, without the use of a magnetic stir bar or the addition of other chemicals to monitor or promote the fusion of proteoliposomes. We used the wicking method in conjunction with a standard membrane capacitance test and a simple method of proteoliposome preparation that generates a heterogeneous mixture of vesicle sizes. To determine the robustness of this technique, we selected two ion channels that have been well characterized in the literature: CLIC1 and **α**-hemolysin. When reconstituted using the wicking technique, CLIC1 showed biophysical characteristics congruent with published reports from other groups; and **α**-hemolysin demonstrated Type A and B events when threading single stranded DNA through the pore. We conclude that the wicking method gives the investigator a high degree of control over many aspects of the lipid bilayer system, while greatly reducing the time required for channel reconstitution.

## Introduction

Planar lipid bilayers (PLB) have been used to study the electrophysiological aspects of many types of ion channels since the early 1960's [1], [2], [3], [4], [5], [6]. In spite of the elegance of the PLB technique, numerous technical challenges can significantly complicate experimental design. There is a great deal of variability between different experimental PLB systems with respect to aperture size, lipid composition, electrodes, buffer choice, and type of channel to be studied. Furthermore, obtaining accurate experimental recordings requires a highly skilled investigator to consistently “paint” a unilamellar bilayer over a small aperture connecting two solution chambers.

More subtle difficulties lie in the reconstitution of ion channels into planar lipid bilayers. For instance, the spectrum of different methodologies reported for generating fusible proteoliposomes is highly variable. Two of the most commonly employed protocols include lipid extrusion through a polycarbonate membrane or sonication, which is often reported with ambiguous fixed frequency and temporal ranges. Fusing proteoliposomes into PLB is also non-trivial, traditionally requiring a rotating magnetic stir bar in the chamber where the proteoliposomes are introduced. While the stir bar promotes the fusion of proteoliposomes, it also introduces significant mechanical agitation to the system that increases electrical noise in the output tracings and can cause the PLB to break. Once a stable bilayer is achieved, the investigator frequently faces the further difficulty of long, unpredictable time intervals required for observation of proteoliposome fusion. This unpredictable parameter is often the rate-limiting step in a successful PLB experiment. Many approaches have been developed in an effort to mitigate this variable, including introducing osmoticants in the buffer such as glycerol or urea. These can increase the rate of fusion, but also add chemical complexity to the system [7], [8]. Despite the limitations mentioned above, the PLB technique when properly executed is arguably the most powerful method for studying the biophysics of single ion channels in a controlled environment.

The last decade has seen considerable advances in automation and miniaturization of PLB systems, allowing, for example, automated formation of thousands of PLB over the course of a few hours [9], [10], [11]. Importantly, however, the literature remains sparse with respect to a technique that enables systematic functional membrane protein reconstitution over a range of targets [12]. We sought to fill this void by developing a method that would allow facile, manual reconstitution of membrane protein complexes. We hypothesized that it would be possible to circumvent the waiting time for fusion of proteoliposomes to occur by manually contacting the proteoliposomes to the PLB using a maneuver that we hereafter refer to as a “wicking” stroke. There is precedent for a similar approach to membrane protein reconstitution using purified SNARE proteins, which belong to the fusogenic protein family [13], but we sought to improve the robustness of this fusion-by-contact approach to include polytopic, non-fusogenic integral membrane proteins. We developed the wicking technique in our laboratory using several types of ion channels in conjunction with a simple method of proteoliposome preparation; and then sought to assess the generalizability of the wicking method by reconstituting two commercially available membrane proteins. Our rationale for this was that successful reconstitution and biophysical characterization of these channels would demonstrate the value of this method to other laboratories faced with the challenges of reconstituting novel proteins.

Our channel selection criteria required that both proteins have well-characterized biophysical parameters with considerable corroborated data reported within the context of reconstitution in PLB. These criteria would allow us to compare reconstitution using the wicking method against a *de facto* standard of reported electrophysiological characteristics. Toward this end, we ultimately chose to demonstrate wicking using the chloride selective anion channel, CLIC1, and the homomultimeric DNA conducting nanopore **α**-hemolysin from *Staphylococcus aureus*. While **α**-hemolysin has been reported to spontaneously insert into the PLB in a fast manner, in our hands we have observed this to not consistently be the case. As such, we chose it to demonstrate that the wicking technique could be used as a more systematic and reliable means to enforce **α**-hemolysin insertion on a shorter time scale under our control. Similarly, we chose to demonstrate wicking for CLIC1 because, to the best of our knowledge, it is a non-fusogenic ion channel that has not been reported to insert in a fast manner and has complex biophysical and biochemical requirements for reconstitution.

## Methods

### Preparation of painting lipids

Painting lipids were prepared 8 hours prior to beginning an experiment. In our hands, we have observed that this length of time correlates with the ability to form a stable PLB formation by wicking. We have not however biophysically investigated why this correlation is observed. As such, shorter preparation times may be possible. Twenty five microliters each of phosphatidylethanolamine (PE) and phosphatidylserine (PS) (Avanti Polar Lipids, Alabaster, AL), which are suspended in chloroform at a concentration of 10 mg/ml, were combined, the organic solvent was evaporated under a stream of nitrogen, and the lipids were dried further under vacuum for 15 minutes. PE and PS were used in these experiments because they have been previously described to allow successful reconstitution of both CLIC1 and **α**-hemolysin [11]. We note, however, that diphytanoyl phosphatidylcholine (DPhPC) is used to reconstitute **α**-hemolysin more commonly than PE/PS in the recent literature. For consistency, we chose a uniform lipid preparation for reconstitution of both channels, as we did not anticipate this would interfere with our ability to assess the usefulness of the wicking technique. The dried lipids were reconstituted in 10 **μ**l of n-decane (MP Bio, Solon, OH) to a final concentration of 50 mg/ml and stored at 4**°**C in the dark.

### Preparation of CLIC1 wicking proteoliposomes

The CLIC1 protein used in these experiments, purchased from OriGene (Rockville, MD), is a human recombinant protein containing a Myc-DDK tag and was expressed/purified from 293-HEK cells. Cells were lysed in a buffered solution containing 25 mM Tris-HCl pH 7.6, 150 mM NaCl, 1% NP-40, 1 mM EDTA, 1xProteinase inhibitor cocktail mix (Sigma), 1 mM PMSF and 1 mM Na3VO4 and CLIC1 was purified by affinity chromatography using an anti-DDK antibody. CLIC1 was provided as a frozen sample in a buffered solution of 100 mM glycine, 10% glycerol, 25 mM Tris.HCl, pH 7.3.

Immediately prior to beginning an experiment, lipids for making proteoliposomes were prepared in an identical manner to the painting lipids except that instead of re-suspending the lipids in n-decane, they were re-suspended in 48 **μ**l of bilayer buffer (140 mM KCL, 10 mM Hepes, pH 6.0). The lipid/buffer mixture underwent 3 cycles of flash freezing on dry ice and thawing. Two microliters of purified CLIC1 protein stock (167 **μ**g/ml) was added to the buffer/lipid mixture to give a final protein concentration of 0.0067 **μ**g/**μ**l. Using an 80 kHz bath sonicator (Laboratory Supplies Co, Inc, Hicksbille, NY, model# G112SPIT), the protein/buffer/lipid mixture was sonicated for 90 seconds in a 4**°**C cold room, and placed on ice. We often observed a minor amount of aggregate that settled to the bottom of the sonication vial. For subsequent wicking, the glass brush was dipped into the proteoliposome solution so as to avoid contact with any settled aggregates.

### Preparation of α-hemolysin wicking proteoliposomes

Alpha-hemolysin was purchased from Sigma-Aldrich (catalog #H9395). **α**-hemolysin is purified in its soluble form and provided by the vendor as a lyophilized sample. Upon arrival, it was reconstituted in 250 mM KCl, 25 mM Tris-HCl, pH 8.0 to a concentration of 50 ng/**μ**l. Reconstituted protein was aliquoted and stored at −80°C. Proteoliposomes were prepared in an identical manner to the CLIC1 wicking proteoliposomes. Following **α**-hemolysin reconstitution, activity was recorded with symmetrical ionic solutions (see below) bathing the bilayer. Subsequently, single-stranded DNA (ssDNA), purchased from Integrated DNA Technologies (Coralville, Iowa), was added to the bilayer buffer and channel activity was recorded. Oligonucleotide sequences used were as follows:

1. oligo dT20 – TTT TTT TTT TTT TTT TTT TT
2. GCG ATC GTG CAT CGT CAT GAA CT
3. AAT TGC AAT GGT TGA TCG CAT GCG CTA TAA CTT AGC ATT CTG TCA TGG CAA TAG AAA CG
4. ACT GCA GTG ACT GAT CGC AAC CGT GGT AAT CTA GCT ATC TGT CAC AGC CAT AGA GAT A.

### Experimental system

Bilayer chambers and cuvettes were obtained from Warner Instruments (Hamdon, CT). All cuvettes had a 150 **μ**m diameter aperture. Channel recordings were performed by “painting” the aperture with painting lipids resulting in the formation of a high-resistance seal between the two chambers. For these studies, all voltages were made relative to the ground (*trans)* chamber and the *cis* chamber was connected to the voltage-holding electrode. Current recordings were digitized at 5 kHz and filtered at 800 Hz. All data were collected using an Axopatch 200B amplifier connected to a CV203BU head stage using CLAMPEX 9.2. All analysis was performed with PCLAMP v. 9.2 (Axon Instruments, Foster City, CA).

### Membrane capacitance measurement

Membrane capacitance was measured as described previously using the pClamp software [14], [15]. The test window calculates the values for Cm (membrane capacitance), Rm (membrane resistance), Ra (access resistance), and T ( = RC). The analog/digital conversion sampling rate was set to sample at a rate that allowed sufficient time for propagation of the test pulse for a duration greater than ∼5T, and this was visually estimated from within the capacitance test software window. The test pulse magnitude was set to be as large as possible in each experiment such that no clipping of the pulse waveform was observed in the test software window; while a test pulse signal was also sent via a splitter to a Tektronix 211 oscilloscope to verify the test pulse magnitude, which varied between 20–60 mV.

### The wicking brush stroke

A glass capillary tube was pulled in half using the heat of a Bunsen burner. The extruded portion of one of the capillary tube halves was then subjected to brief exposure to the flame so as to cause the filamentous region of the pulled glass to curl up into a small glass bead (roughly 200 **μ**m in diameter) at the end of the tube. The bead on the glass brush is typically large enough to completely cover the 150 **μ**m aperture in the bilayer chamber, but small enough that the investigator can visualize the point of contact around the aperture. To perform the wicking stroke, the glass brush is dipped into the sonication vial containing the prepared proteoliposomes (using caution not to touch any protein aggregate that may have settled to the bottom), to coat the glass bead. The protein-coated glass bead (brush) subsequently touches down approximately 20 **μ**m from the perimeter of the aperture at 11 o'clock (−30°) to initiate the wicking maneuver. The proteoliposome-coated brush is dragged in a continuous stroke across the previously painted membrane towards 4 o'clock (or ∼120°) of the aperture. Throughout the stroke, a 100° clockwise rotation of the brush exposes a significant portion of the coated brush surface area to the membrane. The wicking stroke ends roughly 100 **μ**m beyond the painted membrane.

### Proteoliposome formation for electron microscopy experiments

For the electron microscopy experiments, the same CLIC1-protein/buffer/lipid mixture for making wicking proteoliposomes described above was used to test a variety of different liposome formation methods. For proteoliposome formation by extrusion, 150 **μ**l of the protein/buffer/lipid mixture was passed ten times through a 400 nm polycarbonate membrane suspended in a lipid extruder (Avanti Polar Lipids, Alabastor, AL) that was heated to 48°C. For proteoliposome generation by sonication, 50 **μ**l of protein/buffer/lipid mixture was sonicated in a glass conical test tube using an 80 kHz bath sonicator (Laboratory Supplies Co, Inc, Hicksville, NY) for times that ranged from 30 seconds to 30 minutes. All proteoliposomes were immediately placed on Formvar Grids (SPI Supplies, West Chester, PA) and dehydrated under a nitrogen stream. Dehydrated proteoliposomes on Formvar Grids were soaked in a 1% Uranyl Acetate (aq) solution for ten minutes and dried. Grids were loaded into a Hitachi H7650 120 kV transmission electron microscope set to acquire at 80 kV using a 16MP SIA camera (Scientific Instruments and Applications, Duluth, GA). Images were analyzed with image J software: vesicle sizes were analyzed in three regions of interest (ROI) of identical size on each image and averaged.

## Results

**Figure 1:**
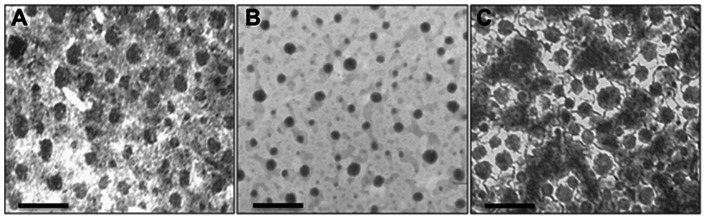
Electron micrographs of proteoliposomes prepared under different conditions. Scale bars represent 200 nm. **A.** Bath sonication for 3 minutes at 80 kHz generated fairly uniform vesicle sizes that averaged 63.4±6.5 nm in diameter. **B.** Lipid extrusion through a polycarbonate filter formed vesicles ranging from 10–50 nm in size with an average diameter of 38.8.±4.2 nm **C.** Bath sonication for only 90 seconds generated a wide range of vesicle sizes ranging from 20–110 nm with a mean diameter of 68.7±14.4 nm.

As a first step in developing the wicking technique, experiments were first performed to define an optimal method of reproducibly preparing a population of proteoliposomes suitable for reconstituting numerous channel types by wicking. We experimented with several types of ion channels and found the most success using proteoliposomes prepared using brief sonication just before beginning an experiment. To more completely characterize this observation, we prepared CLIC1 proteoliposomes for analysis by electron microscopy. The results showed that bath sonication for three minutes at 80 kHz generated a relatively uniform vesicle size that ranged from 50–80 nm with a mean diameter of 63.4±6.5 nm (Figure 1A, n = 3). Proteoliposomes generated by extrusion through a 400 nm polycarbonate membrane generated a population of vesicle sizes that ranged from 10–50 nm, with a mean diameter of 38.8±4.2 nm (Figure 1B, n = 3). Previous experiments in our lab suggested that both of these preparations were sub-optimal for reconstituting some proteins, but not others (data not shown). We report that a 90 second bath sonication at 80 kHz generated a population of vesicles that works well for reconstituting a wide variety of channels by wicking. Interestingly, this sonication time and frequency generated a much larger size distribution of proteoliposomes that ranged from 20–110 nm with a mean diameter of 68.7±14.4 nm (Figure 1C, n = 3).

After preparation of the proteoliposomes, painting lipids prepared 8 hours earlier were used to form a bilayer across a 150 **μ**m aperture using a glass brush. The recording chamber was closed and the membrane capacitance was measured. To determine an empirical membrane capacitance threshold above which we could routinely assume a “transport-competent” bilayer had formed, we modeled the bilayer as a parallel plate capacitor as described previously [14], [15]. Briefly, the capacitance, C, of a pair of plates of a given material can be described as a function of three parameters: the plate area, **A**; the distance, **d**, between the two plates and the dielectric constant, **ε_d_**, of the material from which the plates are made. Hille ascribes an **ε_d_**  = ∼2 for a phospholipid composition where the phospholipid tails are of length 1.2 nm [16]. Using the universal permittivity of free space constant, **ε_0_**  = 8.85×10^−12^ F m^−1^, the plate capacitance can be calculated by the relation:
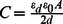


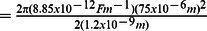


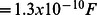



As the bilayer capacitance is historically reported as normalized to unit area, the theoretical bilayer capacitance per unit area is calculated as:
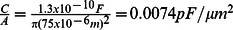



Relative to this range of theoretical values, we sought to find a practical empirical value for the bilayer capacitance per unit area that could be used as a cut-off. As the bilayer thickness, 2d, is inversely proportional to the specific capacitance per unit area, we reasoned that values well above this cut-off could be interpreted as having attained bilayer thickness. In particular, we sought a cut-off that was several orders of magnitude above the theoretical value to account for possible variations in **ε_d_** and A. To arrive at this value, we noted that the average capacitance per unit area was 5.7 pF ±.6 pF/**μ**m^2^ observed over eleven experiments in which we were able to successfully reconstitute CLIC1. Importantly, this does not imply that a smaller value for a cut-off does not exist, but rather that on our system this value was easily attained from “paint” to “paint” and consistently enabled successful reconstitution studies. We also note that the capacitance value of the PLB chamber itself is expected to be on the order of a few pF and contributes to the average capacitance per unit area. However, for the purposes of establishing an empirical capacitance threshold, we found that this contribution can simply be considered as an offset and does not need to be explicitly subtracted. Experimentally, any bilayer whose membrane capacitance value falls below ∼5.7 pF/**μ**m^2^ on our system is considered non-unilamellar and is subsequently ruptured and re-painted. We found that achieving membrane capacitance values above the ∼5.7 pF/**μ**m^2^ threshold can be easily achieved in the first few painting attempts (See Discussion for a thorough description of the possible variations in **ε_d_** and A and the rationale for selecting a cutoff that is several orders of magnitude higher than the theoretical bilayer capacitance value).

**Figure 2:**
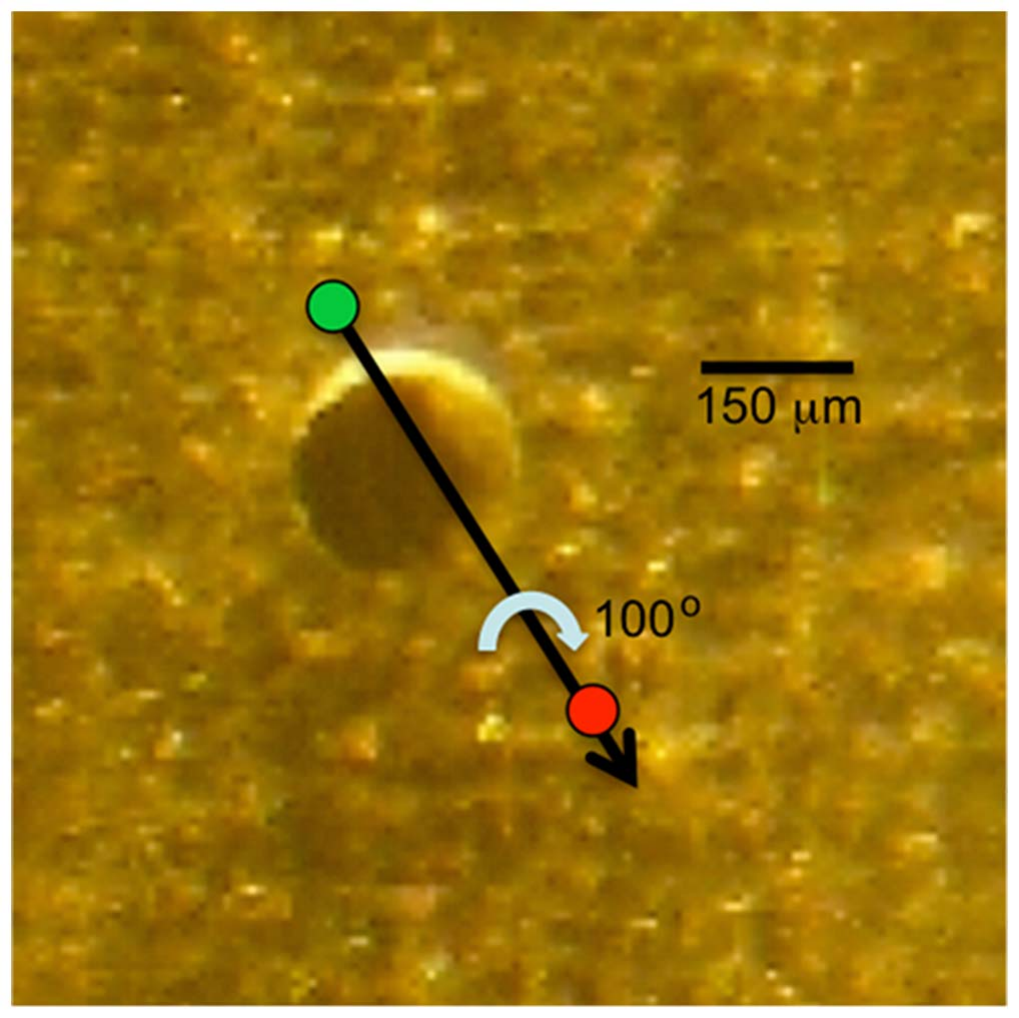
A schematic for the wicking stroke superimposed on an actual image of the aperture with the lipid membrane seen through the microscope. The glass brush coated in proteoliposomes touches down in the general location of the green circle, and initiates the wicking maneuver that ends roughly where the red circle is. The black arrow represents a constant vector for the brush across the two points. Blue arrow shows the clockwise rotation of the brush, which in actual practice is roughly 100 degrees between the two points.

Once capacitance values greater than ∼5.7 pF/**μ**m^2^ are achieved, proteoliposomes can be fused with the bilayer using the wicking stroke. This is performed using a glass brush that has been dipped into the proteoliposome mixture so as to coat the brush head. We have the most success when the wicking brushstroke is performed as described in Methods and illustrated in Figure 2.

**Figure 3:**
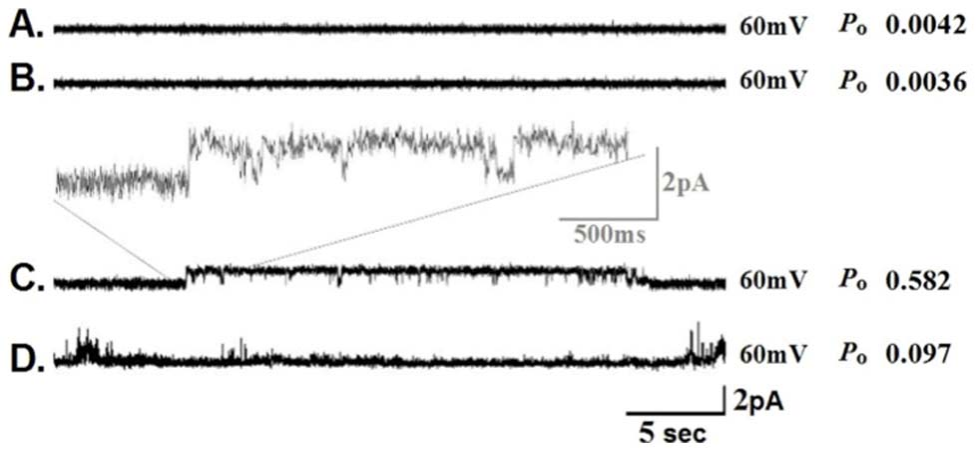
Reconstitution of CLIC1 using the wicking technique. **A**) A thirty-second recording from a lipid bilayer film with a unilamellar thickness that has been verified using the membrane capacitance test. No protein has been wicked onto this bilayer. **B**) A thirty-second recording of the same bilayer as in A, after wicking mock proteoliposomes, which lack any protein. **C**) A thirty-second recording of the same bilayer, wicked with proteoliposomes containing the purified protein CLIC1. Inset shows a magnified portion of the CLIC1 trace exhibiting clear sub-conductive states. **D**) A thirty-second recording of the same bilayer following addition of 50 **μ**M IAA94 on the cis side of the bilayer. All recordings were collected at the indicated holding potential and open probability is reported for each individual trace.

Figure 3 shows representative traces from a typical experiment carried out using the wicking technique and the ion channel, CLIC1. Figure 3A shows a thirty second trace recorded at a holding potential of +60 mV just after the initial bilayer was painted and the thickness verified as unilamellar using the membrane capacitance test. Figure 3B shows a thirty-second trace of the same bilayer, recorded thirty minutes after wicking empty liposomes (liposomes lacking any protein) onto the bilayer. These two traces show almost identical characteristics including virtually non-existent open probabilities, no obvious transitions or gating, and baseline bandwidths of similar amplitude. This demonstrates that wicking empty proteoliposomes does not significantly alter the physical characteristics of the painted bilayer. Figure 3C shows a thirty-second recording taken one minute after wicking CLIC1 proteoliposomes onto the bilayer. Under these conditions, clear state transitions, ionic current, and gating became visible in the trace. These findings suggest that wicking CLIC1 proteoliposomes onto an empty bilayer alters the physical parameters of the membrane to allow ionic current to pass. Over the course of six separate experiments, channel open probability (*P*o) after wicking CLIC1 was 0.568±0.024. This is in reasonably close agreement with previous reports of CLIC1 from inside-out recordings (*P*o = 0.478±0.035) and tip-dip recordings (*P*o = 0.532±0.026) [17].

Next we added a known blocker of CLIC1, [R-(+)-Methylindazone, Indanyloxyacetic acid 94, R-(+)-[(6,7-Dichloro-2-cyclopentyl-2,3-dihydro-2-methyl-1-oxo-1H-inden-5-yl)oxy]acetic acid], known as (IAA94), which has been previously shown to block chloride current through this channel in CHO cells expressing CLIC1; and also in lipid bilayer reconstitution studies [18], [19]. Addition of IAA94 at a concentration of 50 **μ**M to the cis chamber of the bilayer resulted in a reduction in channel gating activity (Figure 3) and a decrease in open probability to 0.141±0.033 (n = 6). Similar levels of blockade have been reported in previous studies of CLIC1 at this concentration of IAA94 [21], [22].

**Figure 4:**
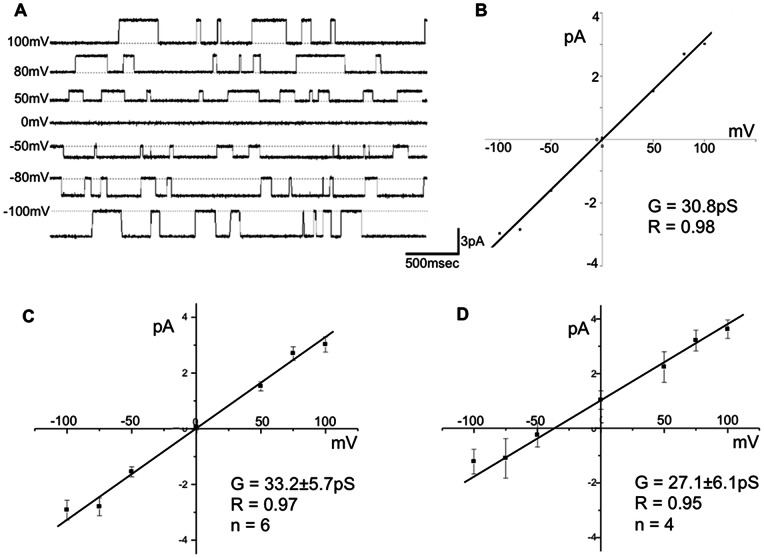
CLIC1 maintains chloride selectivity after reconstitution by wicking. 4A shows single channel current traces obtained at different holding potentials under symmetric concentrations of KCl (140 mM). Traces were later converted into amplitude histograms to calculate current and then plotted against the test voltages. Applying a linear fit to the current/voltage relationship gave a single channel slope conductance of 30.8 pS for this individual experiment (4B). Figure 4C summarizes six of these experiments carried out under symmetrical concentrations of KCl. Figure 4D summarizes experiments conducted under a seven-fold KCl gradient and shows a shift in the reversal potential of −46 mV.

To assess ionic conductance through CLIC1 after wicking onto the bilayer, we recorded single channel traces and analyzed high conductance fast kinetic (HCFK) gating at different holding potentials. Figure 4A shows a series of these recordings obtained in the presence of equimolar (140 mM) KCl on the cis and trans side of the bilayer. When ionic current was plotted against different holding potentials, CLIC1 showed a unitary slope conductance of 33.2±5.7 pS (n = 6). Previous reports have calculated unitary conductance for CLIC1 in CHO cells of 29.6±1.9 pS, and 31.1±1.8 pS in artificial lipid bilayer studies [18], [19], although there is one report in artificial bilayers of CLIC1 conductance as high as 67.5±6.9 pS [17]. Regardless of this discrepancy, these data suggest that wicking does not significantly alter the pharmacological properties, open probability or unitary conductance of CLIC1.

To ensure that the wicking process does not alter the chloride selectivity of CLIC1, experiments were performed in the presence of a seven-fold KCl gradient (140 mM KCl trans, 20 mM KCl cis). Figure 4C shows the current/voltage relationships of CLIC1 under symmetrical KCl concentrations, and gradient conditions (4D). A shift in the reversal potential of 47.6±3.3 mV was observed (n = 4) in the presence of the gradient. This value closely approximates the expected Nernst Potential for chloride under a seven-fold gradient (E_Cl_- ∼51mV) and demonstrates that the primary ionic species traversing the channel is chloride. We conclude that CLIC1 was able to maintain the previously reported chloride selectivity after being reconstituted using the wicking technique.

**Figure 5:**
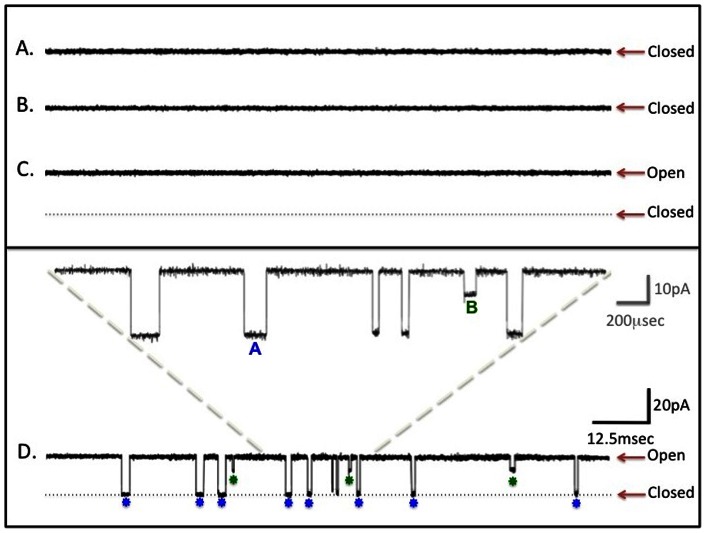
Wicking test of α-hemolysin at a holding potential of +65mV. **A**) Trace recording of a newly painted bilayer in a KCl buffer with no DNA and no proteoliposomes added. **B**) Same bilayer as shown in A, but this time wicked three times with empty liposomes (lacking any protein). **C**) **α**-hemolysin proteoliposomes wicked onto the bilayer shown in A and B. Non-specific current of 23 pA (for this particular trace, red arrow labeled open) is shown, with the dotted line showing zero pA baseline (Red arrow labeled closed). **D**) Trace recording of bilayer shown in C, after 2.5 **μ**M ssdT20 DNA was added to the *trans* chamber. The inset shows examples of five Type A events (Blue) and one Type B event (Green).

To further validate the wicking technique, experiments were performed using the bacterial toxin **α**-hemolysin, a 33 kDa extracellular protein that is secreted by most strains of pathogenic *S. aureus* [20], [21]. We selected **α**-hemolysin supplied from Sigma-Adrich as an additional validation tool because its use has become a common standard for bilayer reconstitution studies [22], [23], [24], [25], [26], [27], [28], [29], [30]. Figure 5 shows traces of an experiment where **α**-hemolysin proteoliposomes were wicked and the membrane potential was then clamped at +65 mV. At this holding potential we consistently observed a 34.6±3.5pS channel (Figure 5C; n = 7). As a means of comparison, reported values in the literature range from ∼60- pS at +60 mV, 80–100 pS at +50 mV and ∼100 pS at +80 mV [24]. Until this point, the only ions in the buffer were potassium, chloride and protons in symmetric concentrations in the *cis* and *trans* bilayer chambers; meaning that at least one or all of these ionic species could permeate the pore under an applied voltage. Upon addition of 2.5 **μ**M oligo dT20 ssDNA to the *trans* chamber, we observed brief blockades of current (Figure 5D) consistent with what is widely accepted to be DNA transport through **α**-hemolysin [24], [25].

**Figure 6:**
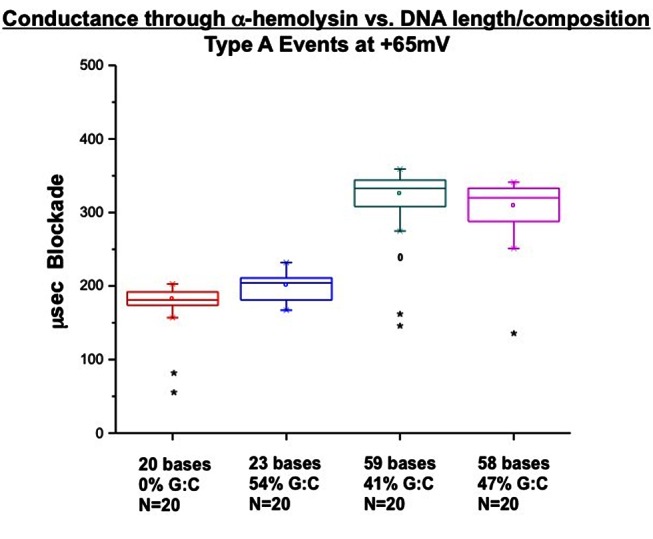
Modified box plots of Type A blockade events using oligonucleotides of different lengths and compositions. Box whiskers represent the maximum and minimum of the statistically significant events in the data sets. Lower and upper bounds of the boxes represent the first and third interquartile values of the data sets. Line within the box is the median of the data. Asterisks are outliers in the data set defined as 3× the Interquartile Range. Degree signs represent suspected outliers defined as 1.5× the Interquartile Range.

Type A and Type B events (see Discussion) were consistently observed (Figure 5D), in agreement with published data [24]. To further demonstrate that the observed blockade of current was resulting from DNA occupancy in the **α**-hemolysin nanopore, additional experiments were conducted using oligonucleotides with varying lengths and compositions. The results of Figure 6 demonstrate that as oligonucleotide length increases, the duration of Type A blockade events increases. Furthermore, there was also an increase in the variation of blockade times within each data set that roughly correlates with the G:C content of the oligo. As G:C pairs have three Watson-Crick hydrogen bonds compared with only two for A:T pairs, any secondary structure as a result of length and composition would presumably alter the kinetics of engagement with the vestibule of the pore and the subsequent occupancy time associated with threading through the **β**-barrel [31]. The dT20 thymidine homomultimers used have the least secondary structure of the four DNA molecules tested and were also the shortest in length. They also exhibited the least variance of any data set and shortest blockade times. Similar studies have concluded that the observed dwell time variations could reasonably arise due to increased complexity of length and secondary structure inherent to the oligonucleotides [31], [32].

Figure 6 also shows the values of suspected and identified outliers, as indicted by “*” and “0”, respectively. As no outliers were observed above the threshold of each data set, we interpret these outliers as possible degradation products of the oligonucleotides, and are included in the modified box plots of Figure 6 for this reason. Taken together, the data suggest that **α**-hemolysin reconstituted by wicking can roughly approximate oligonucleotide length and secondary structure due to composition for single stranded DNA polymers as large as 59 bases.

Finally, to provide a relative, semi-quantitative estimate of the wicking technique against our group's previous method of non-contact fusion by stirring, we note that whereas we observed less than ∼20% reconstitution success rate using non-contact fusion by stirring, we had greater than ∼70% reconstitution success rate by the wicking technique. Importantly, it is crucial to understand that this does not mean that 7/10 wicking strokes ended in successful reconstitution. Rather, it means that 7/10 *experiments* ended in successful reconstitution. To achieve a successful experiment may require numerous wicking strokes, for example as many as ∼100 in some cases. The capacity of this approach for rapid iteration allowed for a drastic increase in experimental success rate. This rapid iteration was enabled by introduction of the capacitance cut-off criteria and the certainty provided by contact insertion. Using the empirical capacitance cut-off, we simply broke the bilayer and repeated the wicking stroke either immediately if the capacitance measurement was below the cut-off or within ∼1 minute if no activity was observed. This allowed us to drastically increase our sampling of the proteoliposome preparation and drastically decrease the time spent waiting for reconstitution. The painting method yielded a comparably much lower success rate of ∼2/10 experiments because we often waited, for example, 4–6 hours for activity without a capacitance cut-off as a guide and extremely limited sampling of the proteoliposome preparation as a consequence of waiting such long periods. It is, however, important to note that a drawback of the technique is that the wicking stroke still requires a skilled operator. We conservatively estimate that the wicking stroke can only be successfully performed roughly every 2 out of 3 times. Despite this limitation, it enabled a several order of magnitude increase in experimental throughput in our hands. Finally, we stress here that the gains to be found in wicking are not truly captured by a measure of successful reconstitution rate alone. Rather, they are to be found in the ability to rapidly break the bilayer if activity is not observed within seconds and re-wick. This process, while it potentially masks the true reconstitution success rate because the bilayer is broken before reconstitution may occur, allows for orders of magnitude higher experimental throughput, which was our initial stated experimental goal.

## Discussion

In this study, we demonstrate that a comprehensive lipid bilayer method we have termed wicking can successfully reconstitute the chloride selective channel CLIC1, and the homomultimeric protein nanopore **α**-hemolysin. We chose these two ion channels because of their rather unusual biophysical properties. CLIC1 has a single pass membrane domain, exists in both soluble and trans-membrane states, and is thought to require auto-oligomerization to form a functional channel [33]. **α**-hemolysin also requires complex assembly in order to realize a functional pore that can thread single-stranded DNA.

High-resolution crystal structure of soluble CLIC1 does not show a protein with any type of pore or channel-like components, which suggests that dramatic conformational rearrangements are required for integration into the membrane [34], [35]. As the wicking stroke physically integrates the protein into the bilayer, it was not clear, *a priori*, that the correct channel conformation could be achieved using this method. Another characteristic of CLIC1 that made it of interest to use as a benchmark for the utility of the wicking technique is its intrinsic property of harboring two states of ionic conductance. These two states have been previously described as slow conductance slow kinetics (SCSK), and high conductance fast kinetics (HCFK) gating [17]. We reasoned that if we could reconstitute CLIC1 successfully and see all of the known functional states of the channel, the technique would likely work for other, simpler channels as well. Although we only present data in this paper that demonstrate HCFK for CLIC1 recordings, we routinely saw the formation of SCSK during the beginning of most successful experiments. As SCSK behavior eventually disappears during the course of an experiment, and is thought to be a minor conductive state of fully reconstituted CLIC1, we chose to focus our analysis only on HCFK. In this sense, CLIC-1 represents a rigorous standard for validation.

The recordings of CLIC-1 activity shown in Figure 3 are complex and reflect SCSK, HCFK, and sub-conductive states. In contrast, the recordings depicted in Figure 4 clearly show single channel activity obtained later in the experiment when SCSK activity has been reported to subside (see Tulk et al) [18]. These traces, and others like it, allow us to compare our measurements of single channel kinetics to the published literature. When analyzed, these data show that CLIC1 reconstituted using the wicking method shows electrophysiological characteristics that are virtually indistinguishable from published findings.

Analogous to those for CLIC1, the requirements of assembly for the **α**-hemolysin pore are complex and also give rise to multiple electrophysiological readouts when conducting DNA. A large number of published reports making use of commercially available **α**-hemolysin (Sigma-Aldrich, catalog #H9395) for lipid bilayer reconstitution studies has made **α**-hemolysin one of a few *de facto* controls to demonstrate competency of a system to support channel reconstitution in PLB [22], [23], [26], [27], [28], [30], [36]. In its secreted form, **α**-hemolysin is soluble and capable of spontaneous insertion into the PLB. Alpha-hemolysin from this commercial source, is commonly reconstituted in both unsupported PLB [22], [23], [26], [27] such as are employed in our system, as well as supported PLB [36]. Because of its use as a *de facto* standard, **α**-hemolysin holds a particularly prominent place in PLB methodology, making its reconstitution via the wicking method of clear use to this community of experimentalists and, potentially, to the community of membrane biologists and biophysicists in general. Moreover, because of its more recent employment as a functionalized sequencing nanopore, application of the wicking technology could be used to increase throughput of reconstitution of functionalized **α**-hemolysin, perhaps paving the way for employment of this method in an industrial device setting [24].

The **α**-hemolysin nanopore requires assembly of single monomeric subunits into a homomultimeric structure consisting of seven subunits. Upon proper assembly of this homomultimeric structure (comprised of an outer vestibule and **β**-barrel inner pore), the **α**-hemolysin complex is known to be capable of ssDNA transport that is described by four unique stereotyped channel transition events referred to in the literature as Type A, B, C and D. Type A events are understood to be the direct translocation of DNA across the membrane, entering the vestibule and exiting through the **β**-barrel. Type B events are indicative of DNA engaging with the vestibule side of the pore but never achieving complete translocation through the **β**-barrel, thereby leading to a transient mid-amplitude blockade of current. Type C and D events are more obscure in the literature and less commonly observed. As such, we chose to focus our analysis on Type A and Type B events, where there is a well-established consensus regarding characterization of biophysical parameters. The fact that we could reconstitute a functional nanopore that possessed Type A and B events demonstrates the robustness of wicking. Furthermore, the ability to detect changes in the recordings that correlate with oligonucleotide length and composition demonstrates that wicking can be used in a molecular device context.

A second component of the method centers on making proteoliposomes rapidly and with little effort. The empirically determined 90-second bath sonication has been successfully employed in our laboratory to reconstitute whole membrane preparations from numerous cell types, **α**-hemolysin, a chimeric inward rectifying potassium channel and CLIC1. Although many factors contribute to proteoliposome formation, including lipid composition, temperature, and protein concentration, this simple bath sonication seems to work well for diverse types of membrane proteins. The fact that this preparation generates a wide range of vesicle sizes might explain its success in reconstituting a diverse array of proteins and may also provide some insight into why there is so much variation in the literature regarding proteoliposome formation. If vesicle size is a factor in generating proteoliposomes for some proteins, then it seems logical that reported methods of preparation would be almost as variable as the ion channels themselves. It follows that having a wide range of vesicle sizes in the mixture would result in a broader range of channel types that could be successfully incorporated into them. Importantly, while our approach makes use of a wide range of proteoliposome diameters in an attempt to as generally as possible accommodate reconstitution of channels previously unfamiliar to us, whether very large or very small proteoliposome diameters are required in either the case of CLIC1 or **α**-hemolysin remains to be investigated. With respect to the technique we report here, we wish only to report that a wider distribution of proteoliposome diameters appears to make the technique robust with respect to ion channels of disparate biophysical and biochemical characteristics.

While we did not compare our wicking technique using different brush structures, such as, for example, a teflon loop instead of a glass bulb, given the rapidity with which we observed reconstitution using our technique (on the order of ∼1 minute), the gains to be had in testing these brush structures were, given our stated goal, minimal. As such, we did not pursue different brush structures further.

In our hands, the wicking technique allows the throughput of lipid bilayer experiments to increase by at least two orders of magnitude. Experimentally, as long as the membrane capacitance test indicates that a true unilamellar lipid film has been painted, wicking can proceed immediately. If ionic conductance is not observed within one minute of wicking, a second wicking stroke is applied. In our experience, ionic current is usually visible after just one wicking application once protein concentration has been optimized. Using this approach, one can reliably assess the quality of bilayer formation with respect to unilamellarity, and exert a much higher degree of control over the insertion of channels into the bilayer. This makes the wicking technique perfectly suited for use in laboratories where functional studies of a particular membrane protein complex are conducted. Although biophysical investigations of how a crude, “macrofluidic” wicking stroke is capable of facile channel reconstitution is beyond the scope of this report, the wicking method is, in our view, relevant to future reconstitution systems. Gaining an understanding of the underlying biophysical and topological events surrounding manual insertion of channels may allow for the eventual development of a detergent-free, automated method for massively parallel *native* membrane protein reconstitution in planar lipid bilayers.

## References

[1] Bean RC, Shepherd WC, Chan H, Eichner J (1969) Discrete conductance fluctuations in lipid bilayer protein membranes. The Journal of general physiology 53: 741–757. 5783009 10.1085/jgp.53.6.741 PMC2202875

[2] Hladky SB, Haydon DA (1970) Discreteness of conductance change in bimolecular lipid membranes in the presence of certain antibiotics. Nature 225: 451–453. 5411119 10.1038/225451a0

[3] Antonov VF, Petrov VV, Molnar AA, Predvoditelev DA, Ivanov AS (1980) The appearance of single-ion channels in unmodified lipid bilayer membranes at the phase transition temperature. Nature 283: 585–586. 6153458 10.1038/283585a0

[4] Fujii G, Chang JE, Coley T, Steere B (1997) The formation of amphotericin B ion channels in lipid bilayers. Biochemistry 36: 4959–4968. 9125518 10.1021/bi962894z

[5] Feigin AM, Takemoto JY, Wangspa R, Teeter JH, Brand JG (1996) Properties of voltage-gated ion channels formed by syringomycin E in planar lipid bilayers. The Journal of membrane biology 149: 41–47. 8825527 10.1007/s002329900005

[6] Mueller P, Rudin DO, Tien HT, Wescott WC (1962) Reconstitution of cell membrane structure in vitro and its transformation into an excitable system. Nature 194: 979–980. 14476933 10.1038/194979a0

[7] Woodbury DJ, Miller C (1990) Nystatin-induced liposome fusion. A versatile approach to ion channel reconstitution into planar bilayers. Biophysical journal 58: 833–839. 1701101 10.1016/S0006-3495(90)82429-2 PMC1281030

[8] Tien HT (2000) Membrane Biophysics as viewed from experimental bilayer lipid membranes; Ottova-Leitmanova A, editor. Amsterdam, The Netherlands: Elsevier.

[9] Funakoshi K, Suzuki H, Takeuchi S (2006) Lipid bilayer formation by contacting monolayers in a microfluidic device for membrane protein analysis. Analytical chemistry 78: 8169–8174. 17165804 10.1021/ac0613479

[10] Malmstadt N, Nash MA, Purnell RF, Schmidt JJ (2006) Automated formation of lipid-bilayer membranes in a microfluidic device. Nano letters 6: 1961–1965. 16968008 10.1021/nl0611034

[11] Poulos JL, Jeon TJ, Damoiseaux R, Gillespie EJ, Bradley KA, et al (2009) Ion channel and toxin measurement using a high throughput lipid membrane platform. Biosensors & bioelectronics 24: 1806–1810. 18849158 10.1016/j.bios.2008.08.041

[12] Simonsson L, Gunnarsson A, Wallin P, Jonsson P, Hook F (2011) Continuous lipid bilayers derived from cell membranes for spatial molecular manipulation. Journal of the American Chemical Society 133: 14027–14032. 21786792 10.1021/ja204589a

[13] Hasan N, Corbin D, Hu C (2010) Fusogenic pairings of vesicle-associated membrane proteins (VAMPs) and plasma membrane t-SNAREs – VAMP5 as the exception. PloS one 5: e14238. 21151919 10.1371/journal.pone.0014238 PMC2997805

[14] White SH, Chang W (1981) Voltage dependence of the capacitance and area of black lipid membranes. Biophysical journal 36: 449–453. 7306666 10.1016/S0006-3495(81)84744-3 PMC1327608

[15] Toyama S, Nakamura A, Toda F (1991) Measurement of voltage dependence of capacitance of planar bilayer lipid membrane with a patch clamp amplifier. Biophysical journal 59(4): 934–944. 2065194 10.1016/S0006-3495(91)82308-6 PMC1281261

[16] Hille B (2001) Ion channels of excitable membranes. Sunderland, MA: Sinauer Associates, Inc.

[17] Warton K, Tonini R, Fairlie WD, Matthews JM, Valenzuela SM, et al (2002) Recombinant CLIC1 (NCC27) assembles in lipid bilayers via a pH-dependent two-state process to form chloride ion channels with identical characteristics to those observed in Chinese hamster ovary cells expressing CLIC1. The Journal of biological chemistry 277: 26003–26011. 11978800 10.1074/jbc.M203666200

[18] Tulk BM, Schlesinger PH, Kapadia SA, Edwards JC (2000) CLIC-1 functions as a chloride channel when expressed and purified from bacteria. The Journal of biological chemistry 275: 26986–26993. 10874038 10.1074/jbc.M004301200

[19] Valenzuela SM, Martin DK, Por SB, Robbins JM, Warton K, et al (1997) Molecular cloning and expression of a chloride ion channel of cell nuclei. The Journal of biological chemistry 272: 12575–12582. 9139710 10.1074/jbc.272.19.12575

[20] Prevost G, Mourey L, Colin DA, Menestrina G (2001) Staphylococcal pore-forming toxins. Current topics in microbiology and immunology 257: 53–83. 11417122 10.1007/978-3-642-56508-3_4

[21] Bakas L, Ostolaza H, Vaz WL, Goni FM (1996) Reversible adsorption and nonreversible insertion of Escherichia coli alpha-hemolysin into lipid bilayers. Biophysical journal 71: 1869–1876. 8889162 10.1016/S0006-3495(96)79386-4 PMC1233654

[22] Le Pioufle B, Suzuki H, Tabata KV, Noji H, Takeuchi S (2008) Lipid bilayer microarray for parallel recording of transmembrane ion currents. Analytical chemistry 80: 328–332. 18001126 10.1021/ac7016635

[23] Mach T, Chimerel C, Fritz J, Fertig N, Winterhalter M, et al (2008) Miniaturized planar lipid bilayer: increased stability, low electric noise and fast fluid perfusion. Analytical and bioanalytical chemistry 390: 841–846. 17972068 10.1007/s00216-007-1647-7

[24] Maglia G, Restrepo MR, Mikhailova E, Bayley H (2008) Enhanced translocation of single DNA molecules through alpha-hemolysin nanopores by manipulation of internal charge. Proceedings of the National Academy of Sciences of the United States of America 105: 19720–19725. 19060213 10.1073/pnas.0808296105 PMC2604925

[25] Mathe J, Aksimentiev A, Nelson DR, Schulten K, Meller A (2005) Orientation discrimination of single-stranded DNA inside the alpha-hemolysin membrane channel. Proceedings of the National Academy of Sciences of the United States of America 102: 12377–12382. 16113083 10.1073/pnas.0502947102 PMC1194911

[26] McGillivray DJ, Valincius G, Heinrich F, Robertson JW, Vanderah DJ, et al (2009) Structure of functional Staphylococcus aureus alpha-hemolysin channels in tethered bilayer lipid membranes. Biophysical journal 96: 1547–1553. 19217871 10.1016/j.bpj.2008.11.020 PMC2717257

[27] Oukhaled G, Mathe J, Biance AL, Bacri L, Betton JM, et al (2007) Unfolding of proteins and long transient conformations detected by single nanopore recording. Physical review letters 98: 158101. 17501386 10.1103/PhysRevLett.98.158101

[28] Pastoriza-Gallego M, Oukhaled G, Mathe J, Thiebot B, Betton JM, et al (2007) Urea denaturation of alpha-hemolysin pore inserted in planar lipid bilayer detected by single nanopore recording: loss of structural asymmetry. FEBS letters 581: 3371–3376. 17601577 10.1016/j.febslet.2007.06.036

[29] Roskamp RF, Vockenroth IK, Eisenmenger N, Braunagel J, Koper I (2008) Functional tethered bilayer lipid membranes on aluminum oxide. Chemphyschem: a European journal of chemical physics and physical chemistry 9: 1920–1924. 18704903 10.1002/cphc.200800248

[30] Suzuki H, Tabata KV, Noji H, Takeuchi S (2007) Electrophysiological recordings of single ion channels in planar lipid bilayers using a polymethyl methacrylate microfluidic chip. Biosensors & bioelectronics 22: 1111–1115. 16730973 10.1016/j.bios.2006.04.013

[31] Szoszkiewicz R, Ainavarapu SR, Wiita AP, Perez-Jimenez R, Sanchez-Ruiz JM, et al (2008) Dwell time analysis of a single-molecule mechanochemical reaction. Langmuir: the ACS journal of surfaces and colloids 24: 1356–1364. 17999545 10.1021/la702368b

[32] McNally B, Wanunu M, Meller A (2008) Electromechanical unzipping of individual DNA molecules using synthetic sub-2 nm pores. Nano letters 8: 3418–3422. 18759490 10.1021/nl802218f PMC2906227

[33] Goodchild SC AC, Breit SN, Curmi PM, Brown LJ (2011) Transmembrane extension and oligomerization of the CLIC1 chloride intracellular channel protein upon membrane interaction. Biochemistry 50: 10887–10897. 22082111 10.1021/bi2012564

[34] Harrop SJ, DeMaere MZ, Fairlie WD, Reztsova T, Valenzuela SM, Mazzanti M, et al (2001) Crystal Structure of the Soluble Form of the Intracellular Chloride Channel NCC27 (CLIC1) at 1.4Å Resolution. Journal of Biological Chemistry 276: 44993–45000. 11551966 10.1074/jbc.M107804200

[35] Goodchild SC Howell MW, Littler DR, Mandyam RA, Sale KL, Mazzanti M, et al (2010) Metamorphic response of the CLIC1 chloride intracellular ion channel protein upon membrane interaction. Biochemistry 49: 5278–5289. 20507120 10.1021/bi100111c

[36] Vockenroth IK, Atanasova PP, Jenkins AT, Koper I (2008) Incorporation of alpha-hemolysin in different tethered bilayer lipid membrane architectures. Langmuir: the ACS journal of surfaces and colloids 24: 496–502. 18085805 10.1021/la7030279

